# PAIPline: pathogen identification in metagenomic and clinical next generation sequencing samples

**DOI:** 10.1093/bioinformatics/bty595

**Published:** 2018-09-08

**Authors:** Andreas Andrusch, Piotr W Dabrowski, Jeanette Klenner, Simon H Tausch, Claudia Kohl, Abdalla A Osman, Bernhard Y Renard, Andreas Nitsche

**Affiliations:** 1 Highly Pathogenic Viruses (ZBS1), Robert Koch Institute, Berlin, Germany; 2 Bioinformatics Unit (MF1), Robert Koch Institute, Berlin, Germany; 3 National Public Health Laboratory, Karthoum, Sudan

## Abstract

**Motivation:**

Next generation sequencing (NGS) has provided researchers with a powerful tool to characterize metagenomic and clinical samples in research and diagnostic settings. NGS allows an open view into samples useful for pathogen detection in an unbiased fashion and without prior hypothesis about possible causative agents. However, NGS datasets for pathogen detection come with different obstacles, such as a very unfavorable ratio of pathogen to host reads. Alongside often appearing false positives and irrelevant organisms, such as contaminants, tools are often challenged by samples with low pathogen loads and might not report organisms present below a certain threshold. Furthermore, some metagenomic profiling tools are only focused on one particular set of pathogens, for example bacteria.

**Results:**

We present PAIPline, a bioinformatics pipeline specifically designed to address problems associated with detecting pathogens in diagnostic samples. PAIPline particularly focuses on userfriendliness and encapsulates all necessary steps from preprocessing to resolution of ambiguous reads and filtering up to visualization in a single tool. In contrast to existing tools, PAIPline is more specific while maintaining sensitivity. This is shown in a comparative evaluation where PAIPline was benchmarked along other well-known metagenomic profiling tools on previously published well-characterized datasets. Additionally, as part of an international cooperation project, PAIPline was applied to an outbreak sample of hemorrhagic fevers of then unknown etiology. The presented results show that PAIPline can serve as a robust, reliable, user-friendly, adaptable and generalizable stand-alone software for diagnostics from NGS samples and as a stepping stone for further downstream analyses.

**Availability and implementation:**

PAIPline is freely available under https://gitlab.com/rki_bioinformatics/paipline.

## 1 Introduction

Next generation sequencing (NGS) has become increasingly popular in the field of diagnostics (Gullapalli *et al.* 2012; Lefterova *et al.* 2015), including pathogen diagnostics. Because of its underlying principle of capturing all nucleic acids in a sample, NGS permits an open view into the sequenced sample and allows screening for any nucleic acids associated with any organism in the sample.

This is especially important for the pathogen detection in a sample because it enables the detection of common and expected, as well as unexpected pathogens and can even serve as a stepping stone in the reconstruction of genomic sequences of hitherto unidentified organisms, provided they are at least somewhat similar to organisms in the used reference databases (Datta *et al.* 2015; Lecuit and Eloit 2014). Due to this fact, NGS is able to overcome the limitations of specific PCR assays, which form the backbone of molecular diagnostics today.

Sequencing libraries generally reflect the DNA composition of a sample rather accurately apart from known biases (Head *et al.* 2014; van Dijk *et al.* 2014), meaning that they contain more fragments from larger or more abundant DNA molecules than from underrepresented ones. The reads generated by the sequencing process essentially mirror this library composition. Genomes of pathogens, specifically viruses, are usually smaller by several orders of magnitude than the genomes of their eukaryotic hosts. Therefore, due to the abundance and size relations of host and pathogen nucleic acids in clinical samples, pathogen-related reads will be hidden among a large number of host-related reads (Marston *et al.* 2013; Tausch *et al.* 2015). This is particularly true for samples with low pathogen loads. Host and pathogen reads in such samples are also commonly called background and foreground reads, respectively.

Metagenomic profiling approaches, which try to estimate the abundances of genomes in a sample using specific reference genome databases, are a common bioinformatical approach to NGS sample composition assessments (Breitwieser *et al.* 2017; Sczyrba *et al.* 2017). Unfortunately, sometimes constituents of a sample that fall below a specific threshold in read count are not reported, often because marginal organisms are irrelevant when analyzing populations for their functional profile or calculating abundance profiles. As a consequence, information about possibly disease-relevant pathogens contained in a clinical sample can be missing. This signifies the importance of identifying organisms reliably based on very few or even individual reads.

Pipelines specifically adapted for pathogen detection generally try to circumvent this problem by reporting every possible hit. Such an increase in sensitivity comes at the expense of specificity, increasing the occurrence of false positive results. These false positives can arise due to random similarities between references contained in the search databases, and the problem is compounded by mutations like single nucleotide variants as well as sequencing errors in the reads. A large amount of false positives can make it difficult to find the true positives among them, thus costing the user valuable time when interpreting the result. This demonstrates the necessity of a good balance between sensitivity and specificity.

However, even when results with the desired accuracy are reached, clinical samples often include a broad spectrum of organisms that are irrelevant to a possible diagnosis. We refer to them as organisms of low interest (OLIs) that may stem from the patient’s natural microbiome, as well as possible contamination sources from the lab or the sequencing platform itself. OLIs can obscure results or obstruct the evaluation depending on their proportion in the results and therefore complicate the identification and validation of important pathogenic agents.

To tackle these problems, we present the program PAIPline (recursive acronym for ‘PAIPline for the Automatic Identification of Pathogens’). It approaches the problems mentioned before by:

- including user-adjustable parameters, with defaults optimized for the discovery of pathogens based on few or even individual reads, achieving the highest sensitivity possible for the algorithm,
- implementing a filtering step that removes reads with low sequence composition complexity that can lead to ambiguous, biologically insignificant read assignments to increase the specificity of the results,
- and providing a list of OLIs, which can be adjusted by the user according to additional metadata of the sample and is used to mask the OLIs in PAIPline’s reports to further unclutter the results.

Moreover, the user-friendly reports include extensive information on all identified operational taxonomic units (OTUs) and can be sorted and filtered based on read counts and taxonomic information. It is worth noting that PAIPline is not limited to any group of pathogens, but can detect all pathogens present in the used databases in parallel. Furthermore, all reads assigned to an OTU are output as a group in a way that makes follow-up validation or analysis particularly easy, for example PCR confirmation in the lab. Additionally, we provide a simple application programming interface (API), which can be used to implement other read assignment methods, allowing PAIPline to be easily extended. Finally, PAIPline is easily installed and comes with no external dependencies except for Python and the applied mapping or alignment tools, which are Bowtie 2 and BLAST (Camacho *et al.* 2009; Langmead and Salzberg 2012).

To assess the performance of PAIPline, it was benchmarked against a selection of previously published and commonly used tools: Pathoscope 2.0 (Hong *et al.* 2014), Kraken (Wood and Salzberg 2014) and Sigma (Ahn *et al.* 2015) on four published biological samples (Kohl *et al.* 2015) and an artificial one. These tools were selected because they are well-known in the field of bioinformatics and are not inherently limited in terms of detectable pathogens by their approach (Forbes *et al.* 2017). The sample selection was made to represent the diversity of samples accurately that can occur in a setting where PAIPline should be used.

In addition to the benchmark, PAIPline was applied to analyze a diagnostic sample within the scope of the public health duties of the Robert Koch Institute and in an international cooperation with the National Public Health Laboratory. This previously published sample originated from an outbreak of hemorrhagic fever cases of then unknown etiology in Sudan in 2014 (Kohl *et al.* 2016). The results of this additional analysis are also presented in this article.

## 2 Materials and methods

**Fig. 1.**
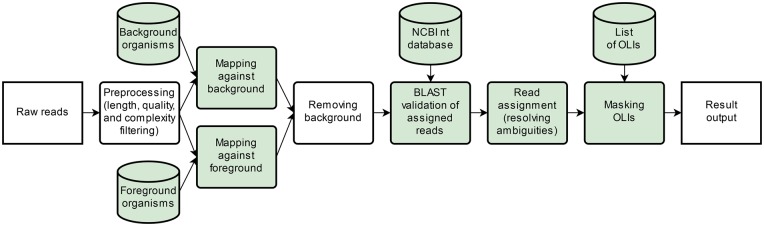
PAIPline standard workflow: The PAIPline for Automatic Identification of Pathogens. Items colored in green indicate user-adjustable parameters or input. First, raw reads are preprocessed, including filters for read length, base quality and read composition complexity. The processed reads are then mapped against user-designated fore- and background databases. The mappings are matched to remove reads originating from background organisms. All remaining read hits are validated by BLAST using the NCBI nt database. Afterwards ambiguities are resolved and the final read assignment is set. Organisms of low interest (OLIs) are then masked, before the final result is presented

The main steps of PAIPline are data preprocessing, read assignment and result evaluation. Below, we describe these steps below along with the sample and data preparation. The complete workflow is visualized in Figure 1.

### 2.1 Preprocessing

The workflow starts with the preprocessing of a set of raw reads in fastq format. Initially, the read input quality control is performed in three steps, the base quality control step, the sequence complexity control step and the length cut-off step.

The base quality control follows a sliding window approach (by default, the window size is 20 and the minimum average quality is Q10). This is done to prevent misleading low-quality bases from contributing to the sensitive alignments which provide the basis for the sample constituents calculation later on.

Subsequently, the remaining bases of the read are checked regarding their sequence complexity based on the SDUST algorithm which discards regions of low complexity and strongly biased composition from the reads (Morgulis *et al.* 2006). This prevents mathematically valid alignments of low biological significance which could possibly stem from regions such as naturally occurring repeat regions. Such regions can be found in different clades and species throughout the tree of life, thus the resulting alignments do not provide insight into the origin of a read. To our knowledge, this is the only pipeline where such a complexity filtering approach for reads is implemented.

Reads are discarded if they are shorter than a minimum length cut-off (36 by default) after the previous trimming steps since such short alignments are extremely unlikely to be unambiguous. All preprocessing parameters can be changed by the user to account for their specific experimental requirements and data.

### 2.2 Read alignment and validation

Following preprocessing, the reads are mapped to the chosen foreground and background databases using Bowtie 2 with its very sensitive mode (Langmead and Salzberg 2012; Langmead *et al.* 2009). Typical databases that can be used with PAIPline are viral, bacterial, fungi, amoebozoa or apicomplexa databases. These can be created using the database updater script described in the subsection Database preparation.

Reads that map to the background are removed from the further analysis, whereas the remaining foreground aligned reads undergo BLAST validation by being queried with the blastn program against the complete NCBI nt database (Altschul *et al.* 1990; Camacho *et al.* 2009). This ensures that for every read all possible origin sequences are found, as long as these are known and included in the NCBI nt database. The user can control the parameters of the applied assignment methods according to his or her needs.

### 2.3 Result presentation

After read assignment, PAIPline generates a result overview. It constructs a taxonomic tree including all OTUs hit by any number of reads and their respective ancestors up to the taxonomic root. Subsequently, this tree is checked for ambiguities by evaluating the hits on each taxonomic rank. A hit is deemed unique if it is only assigned to references within a single OTU. If a read hits several OTUs, this hit is assumed to be unambiguous if the identity to a reference within the best hit OTU is higher than every hit on any other OTU. The identity cut-off for this step can be configured by the user for any named taxonomic rank such as species, genus, family, etc. and reasonable default values are provided. If none of the hits qualify as sufficiently unambiguous, the hits are moved upwards in the tree and compared again on the next-higher rank. Therefore, PAIPline uses a modified lowest common ancestor (LCA) approach (Huson *et al.* 2007). At this point, all user-designated OLIs are marked for filtering purposes.

Afterwards, the constructed taxonomic tree is transformed and saved in a csv file that allows easy parsing, filtering and visualization using third-party applications such as spreadsheet software. The resulting file contains all OTUs, their taxonomic lineage, as well as their respective unique, unambiguous and total hit counts.

### 2.4 Database preparation

Because PAIPline needs databases containing foreground and background organism-associated sequences, we provide an auxiliary script that allows users to download and maintain a local copy of the NCBI nucleotide (nt) database as well as sub-databases of interest. The script downloads the nt database along with the taxonomic information provided by NCBI and re-annotates the contained sequences with their taxonomic lineage, keeping the original NCBI annotation. Afterwards, user-definable sub-databases of taxonomic clades relevant to a pathogen search, for example viruses, bacteria, fungi, apicomplexa and amoebozoaare created along with background databases for host organisms and artificial sequences. Finally, all newly created databases are indexed for use with the alignment tools applied in the workflow of PAIPline. This precomputation has to be done only once and is usable for all PAIPline runs afterwards. The database update script is available under https://gitlab.com/rki_bioinformatics/database-updater.

### 2.5 Benchmarking

To assess the performance of PAIPline, it was benchmarked along a selected set of other previously published metagenomics tools. The benchmarking included Pathoscope 2.0 (Hong *et al.* 2014), Kraken (Wood and Salzberg 2014) and Sigma (Ahn *et al.* 2015) applied on four published biological samples (Kohl *et al.* 2015) and an artificial one. These tools were selected because they are well-known in the field of bioinformatics and are not inherently limited in terms of detectable pathogens by their approach (Forbes *et al.* 2017).

For the evaluation, the precision P was calculated, which is defined as P=TP/(TP+FP), where TP are true positives and FP are false positives. Furthermore, the recall R, defined as R=TP/(TP+FN), where FN denotes false negatives, was evaluated. Lastly, the F-score F1 was determined, which is the harmonic mean between recall and precision F1=2/((1/R)+(1/P)) and is used to average the two during the evaluation.

The datasets used in the benchmarking of PAIPline and the other metagenomic profiling tools were obtained in two ways and were selected to be representative for biological samples from different backgrounds, such as different lab or clinical samples. An artificial dataset was generated using pIRS with its default settings for 100 bp long Illumina reads (Hu *et al.* 2012). It was designed to test the pathogen detection capabilities of all employed tools against typical virus sequences with different degrees of similarity to human genome sequences. The resulting composition of the artificial sample can be seen in Table 1. The biological samples were acquired and sequenced as described previously (Kohl *et al.* 2015). One of these samples was obtained from a marmoset that died from a Sendai virus infection. The other sample used in that study was obtained by infecting fertilized chicken eggs with low doses of Vaccinia virus, an Orthoreovirus, an Influenza virus and a Sendai virus, respectively, to represent a metagenome containing various viruses. From that study the chicken DNA library, chicken RNA library, marmoset DNA library and marmoset RNA library were used for the benchmarking.

**Table 1.** References, their accession numbers and the number of reads simulated from them to form the artificial sample used in this study

References	Accession number(s)	Reads simulated
Homo sapiens	CM000663.2 - CM000686.2, J01415.2	1000000
Human Herpesvirus 1 (HSV-1)	X14112.1	180
Cowpox virus (CPXV)	AF482758.2	104
Human Immunodeficiency Virus 1 (HIV-1)	AF033819.3	87
Yellow fever virus (YFV)	X03700.1	24
Human Adenovirus B2 Type 11 (HAV-11)	AY598970.1	12
Sum		1000407

All analyses were run on an on-site server with 24 cores of 2.2 GHz and 128 GB RAM running Ubuntu 14.04.3 LTS. All tools were run with their respective default parameters except for the number of threads, which was set to 8, where possible. The databases for PAIPline and Pathoscope were created from the NCBI nt database and both tools were provided sub-databases containing the respective background organisms. The database for Sigma was provided by running the included database creation script. For Kraken, the MiniKraken 2014 database was downloaded from https://ccb.jhu.edu/software/kraken/.

### 2.6 Preparation of Sudan sample

The previously published Sudan sample (Kohl *et al.* 2016), that was used in this study, was one of 28 human serum samples. It was inactivated in Qiagen Buffer AVL and extracted using the Qiagen QIAamp Viral RNA Mini Kit, followed by a DNA digest using the Thermo Fisher TURBO DNA-free Kit. The library was prepared using an Illumina Nextera XT DNA Library Preparation Kit and was sequenced on the MiSeq platform.

## 3 Results

### 3.1 Recall

Table 2 shows the PCR-validated pathogens that each program was able to recall in each of the examined samples. For Sigma the end result, which is the abundance estimation, is shown as well as the results generated after the intermediary mapping part of the program. Pathoscope is the only tool to find Yellow fever virus in the artificial sample. On the other hand, PAIPline is the only tool to identify Sendai virus in the Marmoset RNA sample successfully. It also finds reliably the RNA viruses Sendai virus and Influenza A virus in the RNA library preparations while not finding false positives in the DNA preparations of the same samples. Vaccinia virus as a DNA virus is found in both Chicken sample preparations.

**Table 2.** Taxons recalled on species level by the benchmarked programs in the respective datasets

Host	Library	Expected virus	PAIPline	Pathoscope	Kraken	Sigma (Mapping)	Sigma (Abundance)
Chicken	DNA	Vaccinia virus	Yes	Yes	No (Cowpox)	No (Canarypox)	No
		Sendai virus	No	No	No	Yes	No
		Influenza A virus	No	No	No	Yes	No
Chicken	RNA	Vaccinia virus	Yes	Yes	No (Cowpox)	Yes	No
		Sendai virus	Yes	Yes	No	Yes	No
		Influenza A virus	Yes	Yes	No	Yes	No
Marmoset	DNA	Sendai virus	No	No	No	Yes	No
Marmoset	RNA	Sendai virus	Yes	No	No	Yes	No
Artificial sample		Cowpox virus	Yes	Yes	Yes	Yes	Yes
		HIV-1	No	Yes	No	Yes	No
		HAV-11	Yes	Yes	No	No	No
		HSV-1	Yes	Yes	No	Yes	Yes
		Yellow fever virus	No	Yes	No	No	No

*Note*: Entries in parentheses indicate a detection of a species in the same family. PAIPline is the only tool to detect Sendai virus successfully in the Marmoset RNA, whereas Pathoscope is the only tool to detect Yellow fever virus in the artificial sample.

### 3.2 F-score

**Fig. 2.**
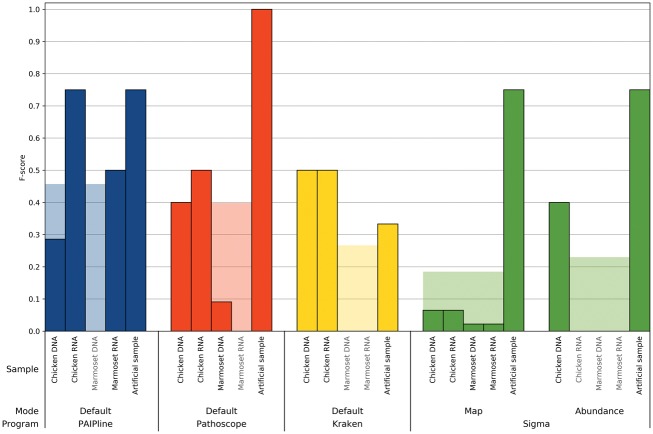
The F-scores on family level for all combinations of samples and benchmarked tools are shown. All tools were run with their default parameters. The transparent bars indicate the mean over all samples processed with that program and mode of operation, whereas light gray sample names indicate no recall. A higher bar generally indicates a better compromise between recall and precision, approximating better real-life performance

**Fig. 3.**
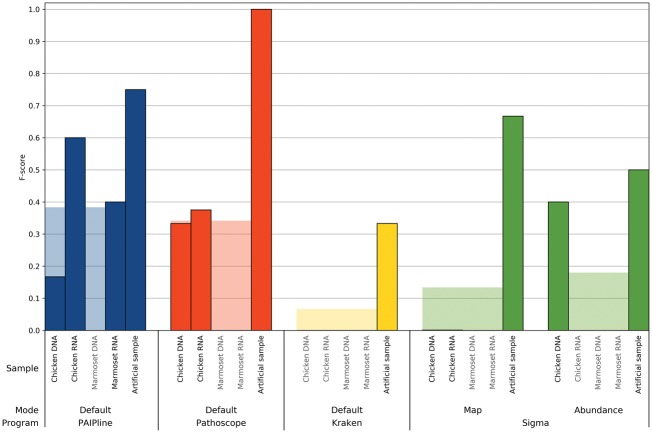
The F-scores on species level for all combinations of samples and benchmarked tools are shown. All tools were run with their default parameters. The transparent bars indicate the mean over all samples processed with that program and mode of operation, whereas light gray sample names indicate no recall. A higher bar generally indicates a better compromise between recall and precision, approximating better real-life performance

Figures 2 and 3 show the F-scores achieved by all tools run with default parameters on all examined datasets on the taxonomic ranks of family and species, respectively. For Sigma, results are shown after the final abundance estimation step as well as after the intermediary mapping step.

### 3.3 Runtimes

**Fig. 4.**
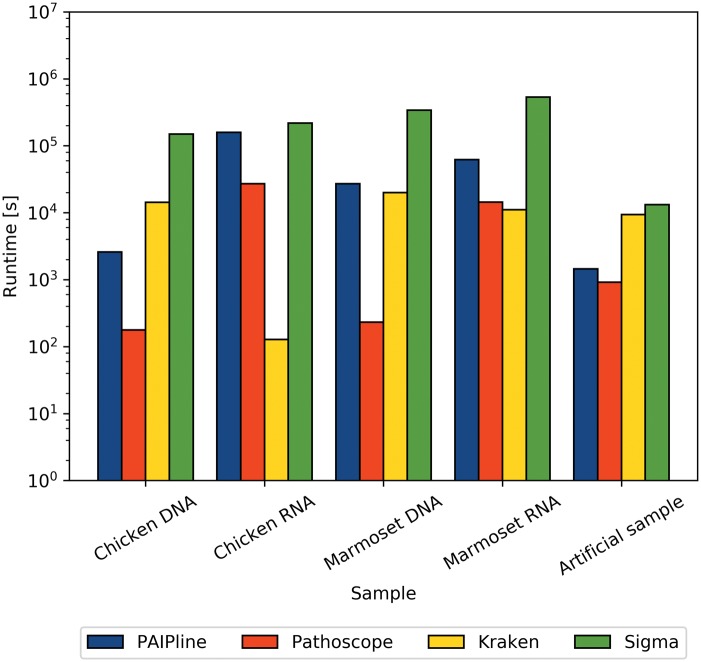
The wall clock times needed to complete each analysis of the given datasets by the benchmarked programs are shown. A higher bar indicates a computationally more expensive or less well parallelized process

A comparison of the runtimes can be seen in Figure 4. It shows runtimes ranging from 10^2^ to 10^6 ^s per run depending on the dataset and respective program. The artificial sample had the lowest average runtimes, with all tools ranging from 10^3^ to 10^4 ^s. Other samples had higher average runtimes as well as more variance between the programs. Sigma generally had the highest runtimes in all samples. PAIPline had the second highest runtimes, except for the artificial and the chicken DNA sample, where it was the second fastest tool. Pathoscope and Kraken were usually the fastest tools, with Pathoscope having the lowest average runtime.

### 3.4 Sudan hemorrhagic fever outbreak data

After the benchmarking, PAIPline was also applied to the Sudan diagnostic sample described above to serve as a real-world application example. Among the results were 45 unambiguously identified reads belonging to Crimean-Congo hemorrhagic fever virus (CCHFV). The CCHFV result was confirmed with PCR. From the domain of the bacteria a prominent finding was 35 unambiguous reads for *Haemophilus parainfluenzae.* However, as of now there was no backtest to validate the bacterial finding in the lab.

## 4 Discussion

PAIPline’s potential as a diagnostic tool was confirmed since PAIPline achieved the best average F-scores compared with other tested tools as seen in Figures 2 and 3. It was also the only tool, apart from the intermediary mapping results of Sigma, to detect reliably the Sendai virus, even though that was the diagnosed causing agent of the marmoset’s respiratory infection. Pathoscope reached the optimal F-score for the artificial sample, but it failed to identify the disease-causing agent in the marmoset samples. This happened even though Pathoscope was provided the same databases as PAIPline, which included the Sendai virus references, and this might be due to default settings for Bowtie 2 during the mapping. Sigma mapped all reads against each of the given references individually, resulting in reads from a distinct biological origin being aligned to several genomes, therefore reporting many hits as false positives after its mapping stage. However, in the abundance estimation phase, almost all species were discarded, thus producing many false negatives. Kraken’s inability to recall anything other than Poxviruses may be due to using the aforementioned MiniKraken database, which might include a smaller number of references than the other used databases, but building a new database was not feasible on our infrastructure due to prohibitive RAM requirements.

Concerning the runtimes, Pathoscope and Kraken are the quickest programs in processing a dataset as seen in Figure 4. This is due to the fact that these programs use the computationally cheapest algorithms out of the tested programs (Langmead and Salzberg 2012; Zielezinski *et al.* 2017). Kraken utilizes an alignment-free approach based on k-mer counts whereas Pathoscope solely relies on a fast mapping with Bowtie 2 and a subsequent read reassignment to produce its results. PAIPline is often the second or third fastest program, which can be explained by the fact that it is the only program to use the computationally expensive, yet sensitive, BLAST algorithm. Sigma also relies on Bowtie 2 to assign an OTU to every read but does extensive downstream calculations for the abundance estimations, thus having the highest runtimes. Three analyses were completed in mere minutes to which extensive use of caching of both references and reads might have contributed.

An important insight from the analysis is that PAIPline failed to find the Yellow fever virus in the artificial sample, which might have happened due to the background substraction step.

Preprocessing or quality control of NGS reads is known to increase the quality and robustness of downstream analyses (Del Fabbro *et al.* 2013). Here, the effect of the preprocessing steps included in PAIPline should be mentioned, as Figures 2 and 3 show that it allows to generate more meaningful biological results. Still, since all samples were run with the same bioinformatical preprocessing settings but stem from different sources and differ in their read qualities, the quality filter has different effects on the samples. Since the artificial sample was simulated with Illumina sequencing characteristics, it has the highest base qualities and therefore seemingly profits least from the quality control. On the other hand, the biological samples in general see an observable increase in their F-scores following the quality control step.

All tools differ greatly in how their results are presented. PAIPline presents results in an easily accessible and processable fashion which was achieved by providing them in a csv format including their taxonomic lineage and hit characteristics. Additionally, OTUs are marked and are filterable based on that marker if they are classified as OLIs. This makes the evaluation of the results easier by providing all relevant information in a condensed form while still permitting deep analysis of the sample constituents. In addition to this, PAIPline provides all unassigned reads in fastq format for possible downstream applications and all positive hits sorted by their OTUs in fasta format for manual validation, for example via online BLAST. This makes it the only tool allowing further scrutiny of the assignment of individual reads, thereby making independent result validation easier.

Along with its good performance on biological and artificial datasets, one of the objectives during the development of PAIPline was to make it as portable and user-friendly as possible. Bioinformatics tools are often difficult to install due to intricate, non-user-friendly setup procedures or out-of-date, unsatisfiable dependencies. Therefore, PAIPline strives to be as independent as possible from external programs and easy to install and use. This is achieved by using only core Python and having two broadly used, popular tools as the only external dependencies.

PCR validation of the results presented above for the diagnostic sample of a patient with a hemorrhagic fever of initially unknown origin has further demonstrated the capabilities of PAIPline as a diagnostic tool. For results to act upon, validation of findings with PCR is suggested. Nevertheless, PAIPline can act as a starting point in a diagnostic pipeline providing an open view on reasons for an infection, including otherwise possibly overlooked organisms.

## 5 Conclusion

In this paper, we show that PAIPline is a viable pathogen identification pipeline for simulated as well as real diagnostic applications and is able to handle common contaminants and present relevant results quickly and accessibly. It is easily installed on most systems and can serve as a stepping stone in diagnostics from NGS samples. PAIPline delivers on average more consistent results, which are better in terms of F-score, across the tested biological and artificial samples than any other tool in our comparison. It delivers solutions to the typical and initially mentioned issues of metagenomic analysis software by implementing meaningful read preprocessing, default parameters tuned for the pathogen detection, validating all possible results, assigning hits to the most reliable LCAs and finally automatically uncluttering reports for a clearer view of the results. Besides providing robust default settings used in this comparison study, PAIPline exposes many parameters that are adjustable to specific experimental settings and thereby offers flexbility to the end user. It can therefore serve reliably as a stand-alone diagnostics software or as a stepping stone for further downstream analyses in the wet and dry lab.

## References

[bty595-B1] Ahn T.-H. et al (2015) Sigma: strain-level inference of genomes from metagenomic analysis for biosurveillance. Bioinformatics, 31, 170–177. 25266224 10.1093/bioinformatics/btu641 PMC4287953

[bty595-B2] Altschul S.F. et al (1990) Basic local alignment search tool. J. Mol. Biol., 215, 403–410. 2231712 10.1016/S0022-2836(05)80360-2

[bty595-B3] Breitwieser F.P. et al (2017) A review of methods and databases for metagenomic classification and assembly. Brief. Bioinformatics. doi: 10.1093/bib/bbx120. 10.1093/bib/bbx120 PMC6781581 29028872

[bty595-B4] Camacho C. et al (2009) BLAST+: architecture and applications. BMC Bioinformatics, 10, 421. 20003500 10.1186/1471-2105-10-421 PMC2803857

[bty595-B5] Datta S. et al (2015) Next-generation sequencing in clinical virology: discovery of new viruses. World J. Virol., 4, 265–276. 26279987 10.5501/wjv.v4.i3.265 PMC4534817

[bty595-B6] Del Fabbro C. et al (2013) An extensive evaluation of read trimming effects on illumina NGS data analysis. PLoS One*,* 8, e85024. 24376861 10.1371/journal.pone.0085024 PMC3871669

[bty595-B7] Forbes J.D. et al (2017) Metagenomics: the next culture-independent game changer. Front. Microbiol., 8, 1069. 28725217 10.3389/fmicb.2017.01069 PMC5495826

[bty595-B8] Gullapalli R. et al (2012) Clinical integration of next generation sequencing technology. Clin. Lab. Med., 32, 585–599. 23078661 10.1016/j.cll.2012.07.005 PMC3479671

[bty595-B9] Head S.R. et al (2014) Library construction for next-generation sequencing: overviews and challenges. BioTechniques, 56, 61. 24502796 10.2144/000114133 PMC4351865

[bty595-B10] Hong C. et al (2014) PathoScope 2.0: a complete computational framework for strain identification in environmental or clinical sequencing samples. Microbiome, 2, 33. 25225611 10.1186/2049-2618-2-33 PMC4164323

[bty595-B11] Hu X. et al (2012) pIRS: profile-based Illumina pair-end reads simulator. Bioinformatics, 28, 1533–1535. 22508794 10.1093/bioinformatics/bts187

[bty595-B12] Huson D.H. et al (2007) MEGAN analysis of metagenomic data. Genome Res., 17, 377–386. 17255551 10.1101/gr.5969107 PMC1800929

[bty595-B13] Kohl C. et al (2015) Protocol for metagenomic virus detection in clinical specimens. Emerg. Infect. Dis., 21, 48–57. 25532973 10.3201/eid2101.140766 PMC4285256

[bty595-B14] Kohl C. et al (2016) Crimean congo hemorrhagic fever, 2013 and 2014 Sudan. Int. J. Infect. Dis., 53, 9.

[bty595-B15] Langmead B., Salzberg S.L. (2012) Fast gapped-read alignment with Bowtie 2. Nat. Methods, 9, 357–359. 22388286 10.1038/nmeth.1923 PMC3322381

[bty595-B16] Langmead B. et al (2009) Ultrafast and memory-efficient alignment of short DNA sequences to the human genome. Genome Biol., 10, R25. 19261174 10.1186/gb-2009-10-3-r25 PMC2690996

[bty595-B17] Lecuit M., Eloit M. (2014) The diagnosis of infectious diseases by whole genome next generation sequencing: a new era is opening. Front. Cell. Infect. Microbiol., 4, 25. 24639952 10.3389/fcimb.2014.00025 PMC3944390

[bty595-B18] Lefterova M.I. et al (2015) Next-generation sequencing for infectious disease diagnosis and management: a report of the association for molecular pathology. J. Mol. Diagn., 17, 623–634. 26433313 10.1016/j.jmoldx.2015.07.004

[bty595-B19] Marston D.A. et al (2013) Next generation sequencing of viral RNA genomes. BMC Genomics, 14, 444. 23822119 10.1186/1471-2164-14-444 PMC3708773

[bty595-B20] Morgulis A. et al (2006) A fast and symmetric DUST implementation to mask low-complexity DNA sequences. J. Comput. Biol., 13, 1028–1040. 16796549 10.1089/cmb.2006.13.1028

[bty595-B21] Sczyrba A. et al (2017) Critical assessment of metagenome interpretation—a benchmark of metagenomics software. Nat. Methods, 14, 1063–1071. 28967888 10.1038/nmeth.4458 PMC5903868

[bty595-B22] Tausch S.H. et al (2015) RAMBO-K: rapid and sensitive removal of background sequences from next generation sequencing data. PLoS One, 10, e0137896. 26379285 10.1371/journal.pone.0137896 PMC4574938

[bty595-B23] van Dijk E.L. et al (2014) Library preparation methods for next-generation sequencing: tone down the bias. Exp. Cell Res., 322, 12–20. 24440557 10.1016/j.yexcr.2014.01.008

[bty595-B24] Wood D.E., Salzberg S.L. (2014) Kraken: ultrafast metagenomic sequence classification using exact alignments. Genome Biol., 15, R46. 24580807 10.1186/gb-2014-15-3-r46 PMC4053813

[bty595-B25] Zielezinski A. et al (2017) Alignment-free sequence comparison: benefits, applications, and tools. Genome Biol., 18, 186. 28974235 10.1186/s13059-017-1319-7 PMC5627421

